# The *PancreasView gemcitabine* transcriptomic signature predicts response to gemcitabine in patients with resected pancreatic ductal adenocarcinoma

**DOI:** 10.1093/oncolo/oyaf083

**Published:** 2025-05-11

**Authors:** Louisa Bolm, Nicolas Fraunhoffer, Nelson Dusetti, Julia Straesser, Natalie Petruch, Carlos Fernandez del Castillo, Juan Iovanna

**Affiliations:** Department of Surgery, Massachusetts General Hospital, Boston, MA 02114, United States; Department of Surgery, University Medical Center Schleswig-Holstein, Campus Lübeck, Lübeck 23562, Germany; Centre de Recherche en Cancérologie de Marseille (CRCM), INSERM U1068, CNRS UMR 7258, Aix- Marseille Université and Institut Paoli-Calmettes, Parc Scientifique et Technologique de Luminy, Marseille 13288, France; Center for Pharmacological and Botanical Studies, Faculty of Medicine, Buenos Aires University, National Council for Scientific and Technical Research, Buenos Aires C1121ABG, Argentina; Centre de Recherche en Cancérologie de Marseille (CRCM), INSERM U1068, CNRS UMR 7258, Aix- Marseille Université and Institut Paoli-Calmettes, Parc Scientifique et Technologique de Luminy, Marseille 13288, France; Department of Surgery, Massachusetts General Hospital, Boston, MA 02114, United States; Department of Surgery, University Medical Center Schleswig-Holstein, Campus Lübeck, Lübeck 23562, Germany; Department of Surgery, Massachusetts General Hospital, Boston, MA 02114, United States; Department of Surgery, University Medical Center Schleswig-Holstein, Campus Lübeck, Lübeck 23562, Germany; Department of Surgery, Massachusetts General Hospital, Boston, MA 02114, United States; Centre de Recherche en Cancérologie de Marseille (CRCM), INSERM U1068, CNRS UMR 7258, Aix- Marseille Université and Institut Paoli-Calmettes, Parc Scientifique et Technologique de Luminy, Marseille 13288, France; Gastroenterology Section, Hospital de Alta Complejidad El Cruce, Florencio Varela, Buenos Aires, 1888, Argentina; School of Medicine, University Arturo Jauretche, Florencio Varela, Buenos Aires, 1888, Argentina

**Keywords:** *PancreasView*, PDAC, transcriptomic signatures, personalized treatment, clinical trial

## Abstract

**Introduction:**

In the current study, we aimed to assess the efficacy of a gemcitabine response predictive signature that is part of the *PancreasView* transcriptomic predictive tool (Gem + or Gem*−*).

**Methods:**

We used a cohort of pancreatic ductal adenocarcinoma patients treated from the Massachusetts General Hospital who underwent upfront resection.

**Results:**

In this cohort of 43 patients, 20 (46.5%) received adjuvant gemcitabine (GEM arm) and 23 (53.5%) did not receive any adjuvant chemotherapy. Among the 43 patients, the *Gem* signature defined a subgroup of 16 patients (37.2%) who were sensitive (*Gem+*) and 27 (62.8%), who were resistant to gemcitabine (*Gem−*). The *Gem+* patients who received adjuvant gemcitabine had significantly better median disease-free survival (DFS) compared to the *Gem−* patients (NR until 72 months of follow-up vs 19.0 months; stratified hazard ratio [HR]: 0.19; 95% CI, 0.04-0.86; *P* = .032) and longer median cancer-specific survival (CSS) (NR until 96 months of follow-up vs 37.0 months; stratified HR: 0.18; 95% CI, 0.04-0.85; *P* = .030) when treated with gemcitabine. The gemcitabine signature remained an independent predictive factor for DFS (HR: 0.41; 95% CI, 0.19-0.89; *P* = .024) and CSS (HR: 0.47; 95% CI, 0.22-1.23; *P* = .059) after adjusting for clinicopathological characteristics in an unstratified univariate Cox hazard model.

**Conclusions:**

This validation of the gemcitabine predictive transcriptomic signature in an independent cohort from Massachusetts General Hospital reinforces the robustness and reliability of this tool. This study highlights the potential of the signature to aid in the personalization of chemotherapy and enhance patient outcomes in pancreatic ductal adenocarcinoma.

**Implications for practice**

The findings of this study suggest that transcriptomic signatures, such as the gemcitabine predictive signature, have the potential to refine chemotherapy selection for pancreatic ductal adenocarcinoma patients. By identifying individuals most likely to benefit from gemcitabine-based therapy, this approach could contribute to more personalized treatment strategies, improving clinical outcomes while minimizing unnecessary toxicity. However, the routine implementation of such predictive tools will require further validation in larger, prospective studies, as well as the establishment of standardized clinical protocols to guide their application.

Integrating transcriptomic signatures into clinical workflows will necessitate a multidisciplinary approach, involving oncologists, pathologists, and bioinformaticians to ensure accurate interpretation and implementation. Additionally, logistical and regulatory considerations must be addressed to facilitate widespread adoption. Future studies should also explore the feasibility of incorporating these signatures into decision-making frameworks for neoadjuvant and metastatic settings, where treatment personalization remains a significant challenge. Ultimately, the successful translation of this approach into clinical practice will depend on demonstrating its reliability, clinical utility, and cost-effectiveness in real-world settings.

## Introduction

Pancreatic ductal adenocarcinoma (PDAC) is a highly aggressive cancer, with only 12% of patients surviving 5 years after diagnosis across all stages.^1^ Only 15% to 20% of patients are candidates for curative-intent surgical resection.^1^ Following upfront surgery, adjuvant therapy is recommended, regimens involve gemcitabine alone or in combination with Nab-Paclitaxel or mFOLFIRINOX (5-fluorouracil, irinotecan, and oxaliplatin). The 5-year disease-free survival (DFS) rate is 26% with mFOLFIRINOX as compared to 19% with gemcitabine, and the median DFS is 21.4 months vs 12.8 months, respectively. However, not all patients benefit from mFOLFIRINOX, and only 20%-30% of patients treated show a treatment response.^2,3^ Furthermore, this combination regimen is associated with high toxicity, with 76% of adverse events in the mFOLFIRINOX group being grade 3 or 4, compared to 53% in the gemcitabine group.^4^ About 30% of patients are not eligible for adjuvant mFOLFIRINOX; in these cases, gemcitabine-based regimens are preferred and recommended by current treatment guidelines.^5^ While the mFOLFIRINOX regimen has become a standard for adjuvant chemotherapy, numerous biomarker-based stratification approaches have been developed in retrospective studies to identify patients who might benefit from adjuvant gemcitabine.

A transcriptomic tool named *PancreasView* was recently published.^5^ This tool was developed by employing a machine learning approach to improve the RNA predictive signatures for gemcitabine, 5-fluorouracil, irinotecan, and oxaliplatin. These improved signatures were validated in a cohort of 343 patients from the PRODIGE-24/CCTG PA6 clinical trial. The signatures appeared to be accurately predictive of a better efficacy in terms of DFS and Cancer-Specific Survival (CSS) in their respective arms. Interestingly, a positive correlation was observed between the number of drugs in the modified FOLFIRINOX (mFFX) regimen to which a tumor was predicted to be sensitive, and both DFS and CSS. Importantly, the signature’s predictions were significantly associated with longer DFS and CSS in patients receiving mFFX and gemcitabine in concordance with the sensitivity predicted by the signatures. By contrast, when patients were not treated according to the signature’s prediction, DFS and CSS were similar to those of patients that show resistance prediction to all chemotherapies.^5^ Remarkably, similar results were observed in metastatic patients.^6^

In the current study, we aimed to assess the efficacy of the *PancreasView* gemcitabine predictive signature by analyzing a cohort of PDAC patients from the Massachusetts General Hospital.

## Methods

### Patient’s cohort description

The current study was approved by the Massachusetts General Hospital Institutional Review Board (IRB approval 2021A000722). The paraffin-embedded tissues (FFPE) from the surgical samples of the patients included in the Massachusetts General Hospital cohort were centralized by an expert pathologist to evaluate the tumor cell amount and determine the areas to be micro-dissected for optimal RNA extraction. All samples had been collected during upfront surgery and before adjuvant chemotherapy. The latest update of the Massachusetts General Hospital trial clinical data was used in this study (2023).

### Patient’s transcriptome profile generation and analysis

QIAGEN AllPrep FFPE kit (Qiagen) was used to extract the RNA for all the samples following the manufactured protocol. From each FFPE block, one 600 μm core was taken centered on neoplastic cells. The quality of FFPE-derived RNA was measured by the proportion of fragments above 200 base pairs (DV200). The RNA library was generated with QuantSeq 3′ mRNA-Seq kit (Lexogen), and the sequencing benefited from equipment and services from the iGenSeq core facility, at the Institut du Cerveau (ICM) iGenSeq platform CHU Pitié-Salpêtrière, with the Illumina NovaSeq 6000 aiming for a minimum of 10 million reads. Raw reads were mapped to the human genome (GRCH38) and the counts were extracted using the Rsubread R package. The RNA counts were normalized by trimmed mean of M-values and log2 transformed.

### Statistical analysis

Overall survival (OS) was calculated from the date of surgery until death. Disease-free survival was calculated from the date of surgery until local or systemic recurrence. Survival curves were estimated using the Kaplan-Meier method. Univariate Cox hazard regression model and Kaplan-Meier curves were computed using the survival R package. The Cox proportional hazard regression model was used for univariate analysis to estimate the hazard ratio (HR) with a 95% CI.

## Results

### Characteristics of the cohort

**Figure 1.**
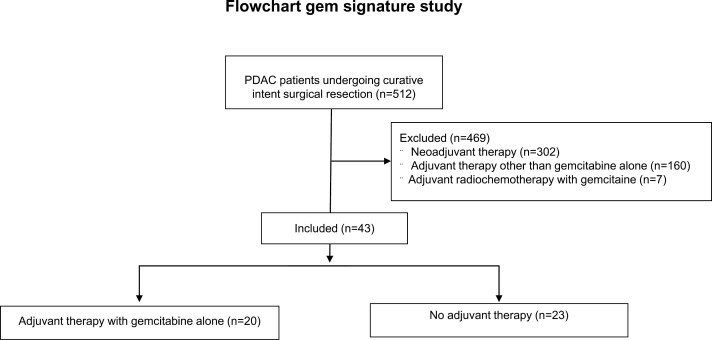
Flowchart showing the distribution and selection of patient’s cohort from Massachusetts General Hospital.

The Gemcitabine signature was assessed using the Massachusetts General Hospital cohort of PDAC patients. Between August 19, 2011, and August 25, 2017, 43 patients were registered in the study as presented in the Flowchart (see Figure 1). An informative sample from the surgical specimen was available for all the 43 patients included in the trial. A sample of the surgical specimen was processed and used for whole-transcriptome profiling using an RNA-sequencing approach. In this Massachusetts General Hospital trial subset, 32 relapses occurred with a median DFS of 9 months. Thirty-four deaths had occurred with a median OS of 25 months. Clinicopathologic characteristics, including treatment group, age, sex, Charlson Comorbidity Index, stage T, lymph node status, histologic grade, resection margins, DFS, and OS were obtained and are presented in Tables S1 and S2.

### Evaluation of the gemcitabine signature in the Massachusetts General Hospital cohort

**Figure 2.**
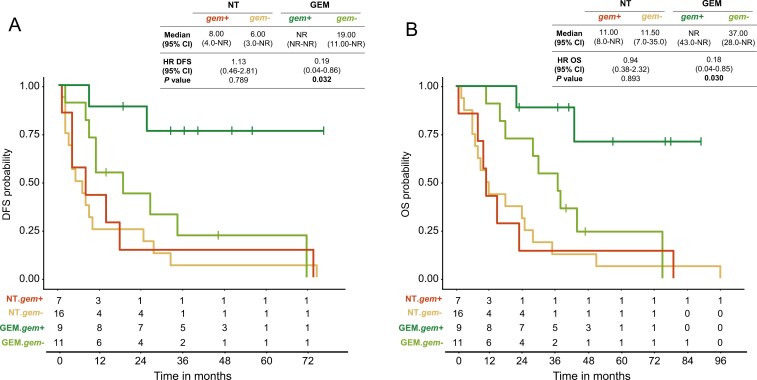
Kaplan-Meier analysis of disease-free survival (A) and overall survival (B) of gemcitabine. DFS, disease-free survival; GEM, gemcitabine arm; HR, hazard ratio; NT, no treated; OS, overall survival.

Out of 43 patients included in the study, 20 (46.5%) received gemcitabine as adjuvant chemotherapy (GEM group) and 23 (53.5%) did not receive adjuvant treatment. Untreated patients showed a shorter DFS (*Gem+* = 8.0 months; *Gem−* = 6.0 months) and OS (*Gem+* = 11.0 months; *Gem−* = 11.5 months) as compared to gemcitabine-treated patients (DFS *Gem+* = not reached (NR) until 72 months of follow-up; *Gem−* = 19.0 months and OS *Gem+* = NR until 96 months of follow-up; *Gem-* = 37.0 months). Among the 43 patients, the gemcitabine signature defined a subgroup of 16 (37.2%) patients who were sensitive to the drug (*Gem+*) and 27 (62.8%), who were resistant (*Gem−*). Gemcitabine prediction did not show a significant association with the clinical variables in the study (Table 1). The *Gem+* patients had a significantly longer median DFS than the *Gem−* patients (NR until 72 months of follow-up vs 19.0 months; stratified HR: 0.19; 95% CI, 0.04-0.86; *P* = .032) and longer median OS (NR until 96 months of follow-up vs 37.0 months; stratified HR: 0.18; 95% CI, 0.04-0.85; *P* = .030) when treated with gemcitabine (Figure 2). Lastly, we evaluated the association between the tumor phenotype determined by PuriST and the gemcitabine signature prediction (Table 1).

**Table 1.** Table 1 summarizes the main clinical and pathological characteristics of the study population. Variables include age, sex, tumor stage, histological subtype, treatment received, and follow-up time.

Signature	*PancreasView* gemcitabine		
Variable	Positive No. events (%)	Negative No. events (%)	*P* value
**Age**			
< 70 yr	6 (37.5)	9 (33.3)	
≥ 70 yr	10 (62.5)	18 (66.7)	1.000
**Sex**			
F	10 (62.5)	13 (48.2)	
M	6 (37.5)	14 (51.8)	.551
**CCI**			
> 5	7 (43.7)	15 (55.6)	
≤ 5	9 (56.3)	12 (44.4)	.665
**Tumor grading**			
1	2 (12.5)	3 (11.1)	
2	9 (56.3)	17 (63.0)	
3	4 (25.0)	7 (25.9)	
Unknown	1 (6.3)	0 (0.0)	.972
**Primary tumor status**			
T1	3 (18.7)	5 (18.5)	
T2	10 (62.5)	19 (70.4)	
T3	3 (18.7)	3 (11.1)	.774
**Nodal status**			
N0	10 (62.5)	16 (59.3)	
N1	6 (37.5)	11 (40.7)	1.000
**Status of surgical margins**			
R0	3 (93.7)	24 (88.9)	
R1	1 (6.3)	15 (11.1)	1.000
**PurIST**			
Basal	1 (6.2)	1 (3.7)	
Classical	15 (93.8)	26 (96.3)	1.000

Abbreviation: CCI, Charlson Comorbidity Index.

## Discussion

The objective of this study was to evaluate the efficacy of a previously reported gemcitabine response predictive signature in a cohort of resected PDAC patients. Specifically, we aimed to validate earlier findings indicating that this signature could predict patient response to gemcitabine-based chemotherapy, with the potential to improve individualized treatment strategies.^5^ To this end, we conducted a comprehensive analysis to assess the predictive performance of the signature in identifying patients who might benefit from adjuvant gemcitabine, as well as those for whom alternative treatments may be more appropriate.

To ensure the robustness of our evaluation, we performed statistical analyses and comparisons with established prognostic markers. We examined OS and DFS in patients stratified by the gemcitabine signature and compared these outcomes to those of patients who received chemotherapy not aligned with their predicted sensitivity. Our findings suggest that the gemcitabine predictive signature could contribute to a more personalized approach to PDAC treatment. The validation of this signature supports its potential role in optimizing treatment selection, potentially leading to improved patient outcomes and reduced exposure to ineffective or unnecessary chemotherapy. The integration of transcriptomic signatures into clinical decision-making may aid in refining adjuvant chemotherapy protocols for PDAC, contributing to a more precise standard of care. Transcriptomic analyses have demonstrated prognostic value in other malignancies, such as breast cancer, where molecular signatures are now incorporated into treatment decision-making, particularly in guiding adjuvant chemotherapy de-escalation strategies.^7^ Given that transcriptomic signatures can capture prognosis, it is essential to establish an objective framework for distinguishing general prognostic factors from treatment-specific responses. For example, previous studies have demonstrated that tumor subtypes, such as basal-like and classical phenotypes, significantly influence patient outcomes and treatment response. Li et al. reported that PurIST stratification could predict response to mFFX, with classical tumors exhibiting better OS than basal-like tumors.^8^ Similarly, O’Kane et al. found that GATA6 expression, a classical subtype marker, was positively associated with response to mFFX.^9^

When applied retrospectively to the phase III adjuvant PRODIGE-24 trial, the gemcitabine signature suggested that approximately 25% of patients received treatments that did not align with their predicted sensitivity, resulting in lower OS (10.8 months), comparable to patients whose tumors were resistant to both treatments (10.6 months). Conversely, patients whose chemotherapy was aligned with the signature prediction exhibited improved OS (33.7 months).^^5^^ These results highlight the potential role of transcriptomic signatures in refining chemotherapy selection to enhance treatment efficacy and minimize unnecessary toxicity. However, the implementation of such signatures in routine clinical practice requires further consideration. While these tools may offer advantages in the neoadjuvant setting by optimizing treatment response and reducing unnecessary toxicity, their role in metastatic PDAC remains less well-defined. Future prospective investigations are needed to determine whether drugs predicted to be effective by transcriptomic signatures maintain efficacy in subsequent treatment lines after tumor progression, as well as whether some therapeutic benefit persists despite a negative signature prediction. Additionally, efforts should be directed toward refining transcriptomic models to better distinguish prognostic influences from treatment-specific responses. Investigating the utility of the signature in metastatic PDAC and assessing its applicability across different treatment lines will provide further insights into its clinical relevance. Lastly, integrating these findings into routine clinical practice will require collaboration with regulatory agencies to establish standardized protocols for transcriptomic-driven treatment selection.

In conclusion, while our study provides evidence supporting the potential of the gemcitabine predictive signature in guiding treatment decisions, further validation in prospective clinical trials is necessary. Additionally, ongoing research should focus on refining predictive models to ensure their applicability across different patient subsets and treatment contexts.

## Limitations of the study

While our findings support the potential utility of the gemcitabine predictive signature, several limitations must be considered:

Retrospective nature of the analysis: The study primarily relies on retrospective data, which may introduce biases and limit the ability to draw definitive conclusions regarding the predictive power of the signature in prospective clinical settings.

Small cohort size: The study is limited by a relatively small number of individuals, which may impact the statistical power and generalizability of the findings.

Lack of validation in independent cohorts: Further validation in larger, independent patient cohorts is necessary to confirm the reproducibility and generalizability of our findings.

Potential confounding effects of tumor heterogeneity: The impact of tumor heterogeneity on treatment response remains a challenge, as additional molecular and genetic factors may influence chemotherapy efficacy beyond the gemcitabine signature alone.

Need for prospective clinical trials: Further prospective studies are required to assess whether transcriptomic signatures retain their predictive value across different treatment lines and whether negative signatures can fully exclude potential treatment benefits.

## Supplementary Material

oyaf083_suppl_Supplementary_Tables_1

oyaf083_suppl_Supplementary_Tables_2

## Data Availability

All raw data supporting the findings of this study are available from the corresponding author upon reasonable written request. Any additional information required to reanalyze the data reported in this paper is also available from the lead contact upon request.
